# Effect of Hip Prosthesis on Photon Beam Characteristics in Radiological Physics

**DOI:** 10.31557/APJCP.2020.21.6.1731

**Published:** 2020-06

**Authors:** Manindra Bhushan, Deepak Tripathi, Girigesh Yadav, Lalit Kumar, Abhinav Dewan, Gourav Kumar

**Affiliations:** 1 *Division of Medical Physics and Department of Radiation Oncology, Rajiv Gandhi Cancer Institute and Research Centre, New Delhi (India)-110085, India. *; 2 *Amity School of Applied Sciences, Amity University (AUUP), NOIDA, India. *; 3 *Dr. APJ Abdul Kalam Technical University, Lucknow (UP), India. *

**Keywords:** Prosthesis, photon, PDD, surface dose, perturbation index

## Abstract

**Introduction:**

Aim of study is to investigate the effect of hip prosthesis on 6 and 15 MV photon beam energies.

**Materials and Methods:**

Prosthesis was kept at the level of tray position. The measurements were done on Varian Clinac-iX linac. Customized prosthesis, termed as Prosthetic Metal Implant (PMI) was made up of wrought austenitic stainless steel rod and covered with paraffin-wax. ‘Standard prosthesis’ was made up of wrought titanium alloy. The dose profiles were measured for three field sizes i.e. 5, 10 and 20 cm at 100 cm SSD for 6 and 15 MV energies. The perturbation index (PI) was also calculated.

**Results:**

Perturbation caused by standard prosthesis was approximately 50% higher than that of PMI. This result may be due to difference in dimension and not because of material composition. Variation of central axis dose might be due to the dimensions of PMI used for experiment which gave intermediate response (e.g. 102.1%, 141.0% and 117.7% for Open, Standard and PMI respectively for 10x10 cm^2^ field size, 10 cm depth and 15MV photon beam setup )as compared to the ‘open’ and ‘standard’ prosthesis. Percentage dose at 10 cm for 6MV photon increased rapidly with field-size for PMI. But, for 15MV photon, difference was not significant. Surface dose (Ds) for PMI remains significantly higher for smaller field.

**Conclusion:**

The perturbation index varied from 0.05 to 0.22 for the measured energies and gave an idea to the planner to assess the behavior of the prosthesis. This range is applicable for both type of implants and for all clinical field-sizes. The attenuation caused by the prosthesis was significant and this effect should be considered in the treatment planning calculations.

## Introduction

Treatment planning of pelvic tumors (like bladder, rectum, anal canal, cervix and prostate) in radiotherapy with high density metallic prosthesis is always a challenging task. The composition of material as well as its form and location needs to be considered by the planner before radiotherapy planning. Behavioral study of high density materials have always been an area of interest in the field of clinical radiation physics. Wieslander and Knoos (2003) investigated the behavior of these materials which may be useful for accurate dose estimation in radiotherapy planning.

The numbers of patients with hip prosthesis are increasing day by day in radiotherapy practice. The position of prosthesis (far or near to the target) alters the dose and creates dose inhomogeneity for the target. Ding and Yu (2001); Tang et al., (2013) reported that in order to avoid this situation, planner should try to avoid the beam entry through prosthesis, but if it is necessary to place the beam in such places, proper estimation of dose is required .

The planning Computed Tomographic (CT) images of patient having high density prosthetic material creates metallic artifacts and hence under- or over-estimation of the radiotherapy dose. The treatment planning systems do not predict the dose accurately for such cases. Monte Carlo simulation is the only way to calculate the dose precisely. The AAPM task group has looked into such scenarios and published a report for understanding the issue scientifically and dosimetrically.

Baxter et al., (2005) concluded that the risk of hip fractures increased substantially in elderly women who had received pelvic irradiation in the past. In case of pelvic fractures and re-irradiation, one should be aware of the nature and behavior of hip prosthesis for proper estimation of the dose. Although the advancement of treatment planning and delivery techniques like intensity modulated radiotherapy (IMRT) has some advantage of static beam angles hereby avoiding metal prosthesis, yet the beam characteristics should be known in presence of a high density material (Peter et al., 2013).

Large number of published literature describe the effect of radiation on pacemakers (Adamec et al., 1982; Katzenberg et al., 1982) and teeth fillings in head and neck cancer patients (Thambi et al. 1979); however not much work has been done to establish the effect of hip prosthesis on photon energies.

Hudson et al., (1984) proposed an assumption of average effective density for the whole prosthesis for calculating distortion in the dose distribution in treatment planning systems. Similarly, Hazuka et al., (1989) noted the dose deviation due to different types of prosthesis in the photon beams and tried to find out the necessary corrections for the same.

In the present study, we investigated the dose distortion caused by the titanium prosthesis and wrought stainless steel prosthesis with different photon beam energies in the water phantom.

## Materials and Methods

The prosthesis was kept at the level of tray position in the collimator of linac head which is the recommended position for the measurements. The experiments were done on Varian Clinac-iX linear accelerator (Varian Medical Systems). This linac is equipped with dual photon energies i.e. 6 MV and 15 MV with additional electron energies (6, 9, 12, 15 MeV). This equipment facilitates the planner to conform the tumour target with Multi-leaf collimator with 120 leaves (characterized by special resolution of 0.5 cm at isocentre for the central 20 cm and of 1.0 cm in the outer 2x10 cm, with a maximum leaf speed of 2.5 cm/sec and a leaf transmission of approximately 1.4%) (Sharma et al., 2014). 

Customized prosthesis, termed as ‘Prosthesis Metal Implant (PMI)’ made up of wrought austenitic stainless steel rod which was then further covered with paraffin-wax to compensate for the tissue effect. Second prosthesis was made up of wrought titanium alloy and was termed as the ‘standard prosthesis’. Dimensions of prosthesis and their experimental setup are mentioned below. As per literature of Mears (1979), the composition of materials in alloy and their densities are mentioned in Table 1.

The dose profiles across the beam were measured in the 3-Dimensional water phantom [Radiation Field Analyser (RFA-300; Blue Phantom, IBA)] using cylindrical chamber CC-13 (IBA) at two depths i.e.dmax and 10 cm in the central axis of beam under the prosthesis. The profiles were measured for three field sizes i.e. 5 x 5 cm^2^, 10 x 10 cm^2^ and 20 x 20 cm^2^ at 100 cm source-to- surface-distance (SSD) for both the energies i.e. 6 MV and 15 MV. The cross-line and in-line profiles were taken and their profile characteristics studied.

In order to report the surface dose, the depth dose curve was plotted using the above experimental setup. Parallel plate chamber PPC40 (IBA Dosimetry, Germany) was kept in the water phantom with source-to-surface-distance (SSD) 100 cm in the central axis and exposure was given for a pre-set number of monitor units i.e. 100 MU. 6 MV and 15 MV beams were used as they are the most usable photon energies in pelvic irradiation. Software ‘OmniPro Accept v7.2 (IBA Dosimetry, Germany)’ was used for the analysis of scanned curves. Depth dose curves were measured upto the depth of 31 cm in water.

The percentage attenuation was calculated using following formula and the perturbation index (PI) was defined as (Sibata et al., 1990):

PI = (A_2_-A_1_)/A_2_

Where, A_1_= Area of dose profile under prosthesis

A_2_= Area of dose profile without prosthesis 

## Results

*Perturbation Index (PI)*

**Figure 1:**
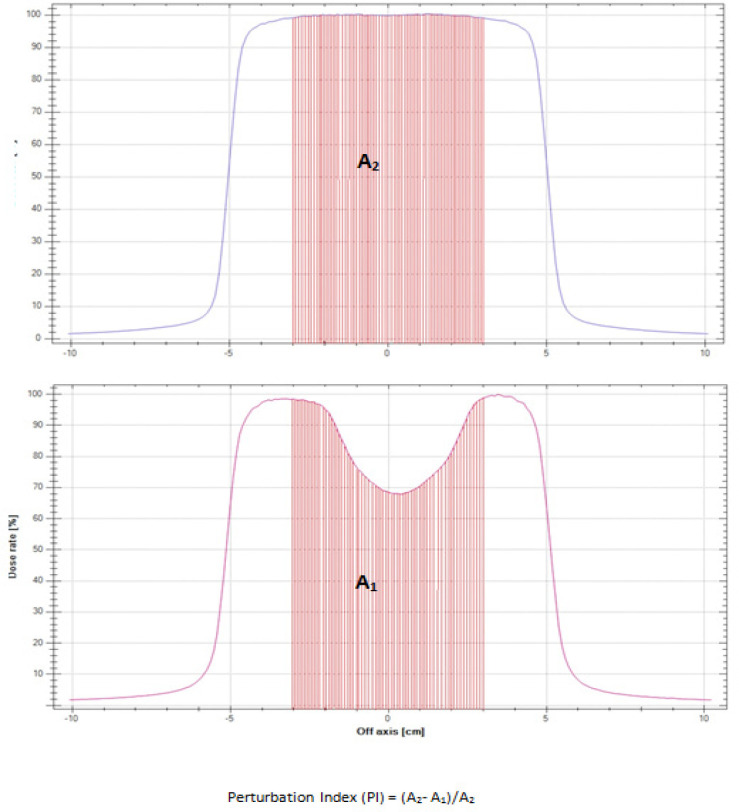
Profile with Perturbation Showing A_1_ and A_2. _Perturbation Index (PI) = (A_2_- A_1_)/A_2_

The area of dose profile under prosthesis i.e. A1, was calculated using integration of data, acquired under the prosthesis Figure 1. The results were analyzed and are tabulated in Table 2. 

*Central Axis Dose Variation (Ddev%)*

The measured data is tabulated in Tables 3 and 4. The beam in open medium delivers a particular dose to a fixed point. But due to the introduction of high density metallic implants, the dose deviates from the above reference point. Central axis dose for standard prosthesis deviates more for 10 x 10 cm^2^ field size for both 6 MV and 15 MV energies in the cross-line profile, in comparison to the PMI and open medium which was also reported by Jia et al., (2015). In-line profile was also measured and the results are tabulated in Table 4. It is clear that there is no significant variation in the central axis dose in this direction. It is evident that dose deviated like 101.1%, 146.9% and 122.0% for open, standard and PMI setup respectively for 5x5 cm^2^ at the depth of dmax for 6MV beam. Similarly, it is 104.1%, 142.5% and 118.7% for open, standard and PMI setup respectively for 20x20 cm^2^ at the depth of 10.0 cm for 15 MV photon beam.

*Percentage Depth Dose (PDDs)*

**Figure 2:**
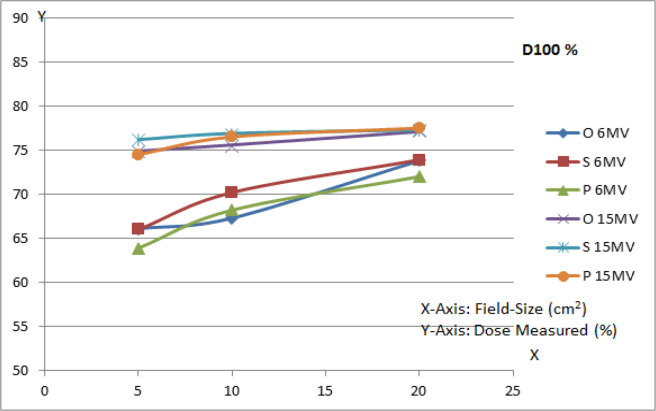
Variation of Dose at 10 cm Depth for Different Photon Energies and Different Prosthesis Setup & Different Field Sizes

The x-ray photon beam attenuates and scatters as per inverse-square law after entering into the patient surface or water medium (Kaushik et al., 2017). The presence of metallic implant in the beam path hardens the beam and thereby increases the mean energy of the beam as evident from the plotted data Figure 2. 

*Surface Dose (Ds)*

**Figure 3:**
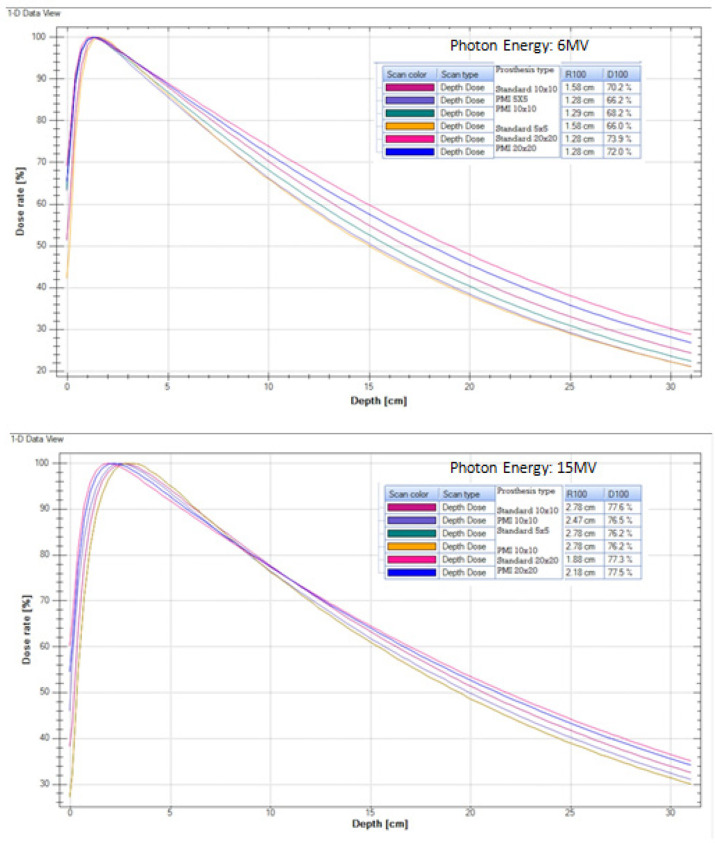
Variation of Percentage Depth Dose (PDD) Curves

Study of ‘surface dose’ parameter is an important aspect in the study of behavior of implant material. In order to find out the changes in surface dose, the PDDs were measured. The data acquired from these profiles was plotted in the form of curve Figure 3. 

**Table.1:** Composition of Prosthetic material. British Standard 3531 (1981) Approved Alloys for Surgical Implants

Principal constituents	Wrought austenitic stainless steel (%)	Wrought Ti alloy (%)
Cr	24.0-26.0	-
Ni	19.0-22.0	-
C	0.08 max	0.08 max
Si	0.75 max	-
Mn	2.0 max	-
P	0.045 max	-
S	0.03 max	-
Mo	0.75 max	-
Cu	0.50 max	-
Fe	Balance	0.25 max
V	-	3.5-4.5
O	-	0.13 max
Al	-	5.6-6.5
Ti	-	Balance
(b) Relative density to water		
Relative density	7.9	4.4
Relative electron density	6.6	3.7

**Table 2:** Measured Perturbation

Energy	Point of Measurement (cm)	Field Size (cm^2^)	Perturbation Index (PI)	
			Ti Alloy	Wrought SS
6 MV	Dmax	5 x 5	0.211	0.09
	10 cm		0.182	0.083
	Dmax	10 x 10	0.195	0.089
	10 cm		0.178	0.074
	Dmax	20 x 20	0.215	0.106
	10 cm		0.172	0.075
15 MV	Dmax	5 x 5	0.178	0.08
	10 cm		0.178	0.076
	Dmax	10 x 10	0.16	0.076
	10 cm		0.153	0.071
	Dmax	20 x 20	0.178	0.065
	10 cm		0.111	0.05

**Table 3:** Analysis of Cross-Line Beam Profile Parameters and Variation due to Prosthesis

Beam Energy (MV)	Field Size (cm^2^)	Depth of Measure-ment (cm)	Prosthesis Setup	Flatness (%)	Symmetry (%)	Field Width (cm)	Penumbra (cm)	Central Axis Dose Deviation (Ddev%)
6	5	1.5	Open (O)	3.8	0.1	5.04	0.53-0.53	101.1
			Standard (S)	19.8	4.3	5.32	0.39-0.44	146.9
			PMI (P)	9.9	0.8	5.22	0.45-0.46	122
		10	Open (O)	4.5	0	5.47	0.61-0.61	100.3
			Standard (S)	17	4.4	5.74	0.48-0.52	139
			PMI (P)	8.6	1.2	5.66	0.53-0.53	118.4
	10	1.5	Open (O)	1.9	0.1	10.18	0.58-0.57	100.7
			Standard (S)	22.6	1.5	10.43	0.43-0.45	156.7
			PMI (P)	2.3	0.3	10.36	0.64-0.63	103.3
		10	Open (O)	3	0.1	11.03	0.71-0.71	100.5
			Standard (S)	19	1.9	11.3	0.67-0.66	145.3
			PMI (P)	2.9	0.1	11.06	0.73-0.71	102.1
	20	1.5	Open (O)	1.8	0.1	20.34	0.60-0.60	100.9
			Standard (S)	22.1	0.6	20.75	0.55-0.55	154.4
			PMI (P)	10.2	0.1	20.58	0.54-0.53	122.6
		10	Open (O)	4.1	0.2	22.01	0.89-0.85	100.6
			Standard (S)	18.1	0.8	22.46	1.14-1.14	142.8
			PMI (P)	8.2	0.1	22.27	0.86-0.87	117.8
15	5	3	Open (O)	4.8	0.4	5.15	0.60-0.59	100.7
			Standard (S)	13.4	3.7	5.37	0.50-0.54	130.3
			PMI (P)	7.3	0.2	5.29	0.55-0.55	115.6
		10	Open (O)	5.4	0.7	5.5	0.66-0.66	100.8
			Standard (S)	12.7	4	5.73	0.57-0.59	126.6
			PMI (P)	6.2	1.2	5.64	0.62-0.60	113
	10	3	Open (O)	2.3	0.3	10.36	0.64-0.63	103.3
			Standard (S)	19.1	1.4	10.59	0.55-0.56	146.5
			PMI (P)	9.2	0.1	10.48	0.57-0.56	120.1
		10	Open (O)	2.9	0.1	11.06	0.73-0.71	102.1
			Standard (S)	17.5	1.3	11.29	0.67-0.67	141
			PMI (P)	8.3	0.5	11.18	0.67-0.65	117.7
	20	3	Open (O)	2.7	0.1	20.22	0.65-0.64	105.1
			Standard (S)	19.5	0.5	21.07	0.64-0.63	147.1
			PMI (P)	9.6	0	20.91	0.64-0.62	121.2
		10	Open (O)	2.2	0	20.22	0.86-0.86	104.1
			Standard (S)	17.9	0.5	22.48	0.84-0.84	142.5
			PMI (P)	8.6	0	22.31	0.78-0.78	118.7

**Table 4:** Analysis of In-Line Beam Profile Parameters and Variation due to Prosthesis

Beam Energy (MV)	Field Size (cm^2^)	Depth of Measure-ment (cm)	Prosthesis Setup	Flatness (%)	Symmetry (%)	Field Width (cm)	Penumbra (cm)	Central Axis Dose Deviation (Ddev%)
6	5	1.5	Open (O)	3.8	0.0	5.1	0.55-0.58	100.7
			Standard (S)	11.1	0.7	5.07	0.56-0.74	103.8
			PMI (P)	4.1	0.4	5.2	0.57-0.60	100.6
		10	Open (O)	4.3	0.0	5.53	0.65-0.67	100.1
			Standard (S)	11.4	0.5	5.51	0.66-0.88	102.9
			PMI (P)	4.4	0.7	5.64	0.66-0.70	100.0
	10	1.5	Open (O)	1.8	0.1	10.25	0.61-0.63	100.8
			Standard (S)	8.6	0.9	10.17	0.62-0.60	102.9
			PMI (P)	1.9	0.4	10.25	0.62-0.66	100.4
		10	Open (O)	3.4	0.1	11.12	0.78-0.79	100.3
			Standard (S)	7.5	0.4	11.06	0.84-0.93	102.6
			PMI (P)	3.7	0.2	11.13	0.82-0.83	100.4
	20	1.5	Open (O)	2.1	0.1	20.47	0.66-0.70	101.1
			Standard (S)	18.9	1.5	20.54	0.68-0.66	124.8
			PMI (P)	12.4	3.3	20.26	2.54-0.61	102.3
		10	Open (O)	4.0	0.1	22.18	0.94-0.96	100.8
			Standard (S)	15.7	1.3	22.62	1.12-1.11	118.2
			PMI (P)	12.2	2.9	21.96	3.77-0.93	100.5
15	5	3	Open (O)	5.5	0.6	5.07	0.62-0.64	100.8
			Standard (S)	10.5	0.0	5.17	0.64-0.82	102.1
			PMI (P)	5.0	0.0	5.3	0.64-0.67	100.7
		10	Open (O)	6.1	0.6	5.41	0.69-0.70	101.0
			Standard (S)	10.6	0.0	5.52	0.72-0.90	101.8
			PMI (P)	5.5	0.8	5.65	0.72-0.74	100.4
	10	3	Open (O)	1.6	0.3	10.42	0.68-0.69	102.3
			Standard (S)	7.3	0.1	10.37	0.69-0.76	105.6
			PMI (P)	1.5	0.2	10.44	0.68-0.71	102.3
		10	Open (O)	2.3	0.1	11.13	0.79-0.79	101.7
			Standard (S)	6.6	0.6	11.08	0.83-0.89	104.0
			PMI (P)	2.7	0.6	11.15	0.81-0.81	101.7
	20	3	Open (O)	3.0	0.4	20.22	0.71-0.72	106.1
			Standard (S)	14.6	1.1	20.89	0.72-0.74	122.9
			PMI (P)	10.3	3.1	20.68	1.59-0.70	106.3
		10	Open (O)	2.2	0.2	22.02	0.86-0.86	104.4
			Standard (S)	13.3	1.0	22.29	0.91-0.89	119.7
			PMI (P)	9.5	2.9	22.06	1.92-0.82	103.5

**Figure 4:**
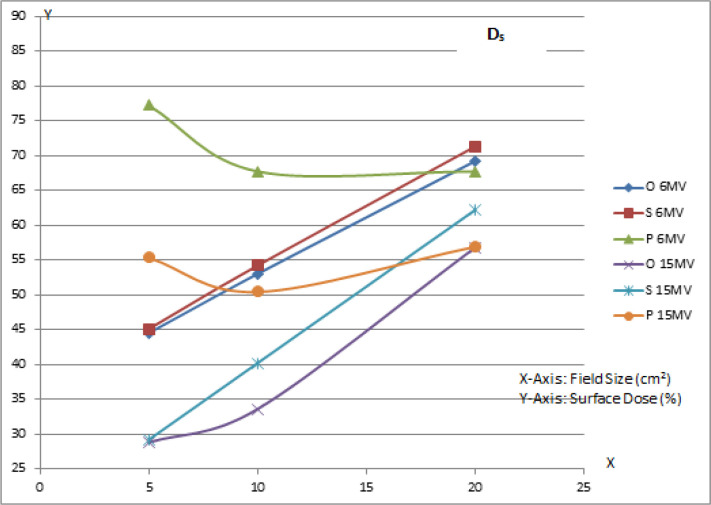
Variation of Surface Dose (D_s_)

## Discussion

Conformity of dose is essential part for any clinically deliverable plan. Dose conformity assures the planner for better clinical outcomes. But, the introduction of any metallic object in the path of the beam has chances to deviate the doses from its original dosimetric values which was also reported by Mohammadi et al., (2017). The effectiveness of treatment depends on the plan made by the planner (medical physicist) and evaluated by radiation oncologist, for delivering optimum treatment to the patient. But the metallic high-Z implants create artifacts in the computed tomographic images and alter the dose distribution. It is necessary for any planner to know the beam characteristics in the presence of such objects for better assessment of the treatment plans.

*Perturbation Index (PI)*

It is evident that the perturbation caused by prosthesis made up of titanium alloy was approximately 50% higher than that made up of wrought austenitic stainless steel. This was due to the difference in dimensions of the prosthesis. It may be noted that the composition of the implant was not accurately determined. Although, it may be possible to measure it inside the laboratory, yet the prosthetic implant inside the patient body cannot be measured accurately by any means. Mahuvava et al., (2018) also found that the absorbed dose dropped significantly due to beam attenuation. For bilateral prosthesis, the target dose was reduced upto 23% and 17% for stainless steel and titanium prosthesis. Also, for unilateral hip prosthesis, the dose reductions were 19% and 12% respectively.

*Central Axis Dose Variation (Ddev%)*

The measured data showed that variation of central axis dose was approximately 20% for PMI material, whereas the standard implant created a 50% variation in central axis dose in comparison to the open field. The reason behind this might be the dimension of PMI used for experiment, which gave intermediate response (mentioned in Tables 3 and 4) as compared to the ‘open’ and ‘standard’ prosthesis. Shimozato et al., (2010) examined the parameter for skull fixation and found the differences between simulation and the planned results on the entrance and exit sides of the plate as 23.1% and 12.7% respectively.

*Percentage Depth Dose (PDDs)*

Percentage dose at 10 cm depth for 6 MV photon increased rapidly with field-size. The metallic implant PMI also showed a similar pattern. But, for 15 MV photon beam, the difference among all the three setups was not significant. This might be possible due to the high linear energy transfer and lesser attenuation in the medium Figure 4. Lesser attenuation of higher photon beam energies deposit maximum dose to any reference point in the medium. The similar pattern was followed in the present study also. Carolan et al., (2000) studied the effect of hip prosthesis and showed a dose reduction of 52% in the shadow of the prosthesis. A Monte Carlo simulation confirmed an increase in dose to the distal surface of the prosthesis by approximately 35%.

*Surface Dose (Ds)*

The curve showed that the surface dose (Ds) for PMI remained moderately higher for smaller fields and showed a gradual decrease and further increase in pattern. This might be possible due to the scatter contribution in the dose output with increasing field size. But, for the standard prosthesis, the curve increased linearly with field size. For open setup, the curve follows a similar pattern, but remains at lower side. Catli (2015) also has demonstrated that the dose increases in the tissue at a distance of 2 mm in front of the implant due to backscatter.

*Limitations*

This study highlights the beam characteristics in the presence of high density metallic implant in the water medium. But the limitation of the present study is its lack of correlation with the clinical data. Hip prosthesis will always be implanted within the body; hence more studies are needed for better understanding of the treatment planning parameters related to the actual patient data.

In conclusion, our study elaborates the available information in the literature regarding different prosthetic materials in different photon energies. The attenuation caused by the prosthesis was significant and this effect should be considered in the treatment planning calculations. The surface dose changed significantly especially for the smaller field sizes and hence was more significant for the intensity modulation. The perturbation index varied from 0.05 to 0.22 for the measured energies which are in contrast with observations made by Sibata CH et al., (1990) and gave an idea to the planner to assess the behavior of prosthetic material used.

## References

[B1] Adamec R, Haefliger JM, Killisch JP, Niederer J, Jaquet P (1982). Damaging effect of therapeutic radiation on programmable pacemakers. Pace.

[B2] Baxter NN, Habermann EB, Tepper JE, Durham SB, Virnig BA (2005). Risk of pelvic fractures in older women following pelvic irradiation. JAMA.

[B3] Carolan M, Dao P, Fox C, Metcalfe P (2000). Effect of hip prosthesis on radiotherapy dose. Australasian Radiol.

[B4] Çatli S (2015). High-density dental implants and radiotherapy planning: evaluation of effects on dose distribution using pencil beam convolution algorithm and Monte Carlo method. J Appl Clin Med Phys.

[B5] Ding GX, Yu CW (2001). A study on beams passing through hip prosthesis for pelvic radiation treatment. Int J Radiat Oncol Biol Phys.

[B6] Hazuka MB, Ibbott MS, Kinzie JJ (1989). Hip prostheses during pelvic irradiation: effects and corrections. Int. J.Radiat. Oncol.Biol. Phys.

[B7] Hudson FR, Crawley MT, Samarasekera M (1984). Radiotherapy treatment planning for patients fitted with prosthesis. Br J Radiol.

[B8] Jia Y, Zhao L, Cheng CW (2015). Dose perturbation effect of metallic spinal implants in proton beam therapy. J Appl Clin Med Phys.

[B9] Katzenberg CA, Marcus FI, Heusinkvield RS, Mammana RB (1982). Pacemaker failure due to radiation therapy. Pace.

[B10] Kaushik S, Punia R, Tyagi A (2017). Dosimetric studies of cadmium free alloy used in compensator based intensity modulated radiotherapy. Radiat Phys Chem.

[B11] Mahuvava C, Du Plessis FC (2018). Dosimetry effects caused by unilateral and bilateral hip prostheses: A monte carlo case study in megavoltage photon radiotherapy for computed tomography data without metal artifacts. J Med Phys.

[B13] Mohammadi K, Hassani M, Ghorbani M, Farhood B, Knaup C (2017). Evaluation of the accuracy of various dose calculation algorithms of a commercial treatment planning system in the presence of hip prosthesis and comparison with Monte Carlo. J Can Res Ther.

[B14] Peter WJ Voet, Maarten LP Dirkx, Sebastiaan B (2013). Automated generation of IMRT treatment plans for prostate cancer patients with metal hip prostheses: Comparison of different planning strategies. Med Phys.

[B15] Reft C, Alecu R, Das IJ (2003). Dosimetric considerations for patients with HIP prostheses undergoing pelvic irradiation. Report of the AAPM Radiation Therapy Committee Task Group.

[B16] Sharma MK, Mitra S, Saxena U (2014). Is volumetric modulated arc therapy (RapidArc) better than intensity modulated radiotherapy for gynecological malignancies? A Dosimetric comparison. J Can Res Ther.

[B17] Shimozato T, Yasui K, Kawanami R (2010). Dose distribution near thin titanium plate for skull fixation irradiated by a 4-MV photon beam. J Med Phys.

[B18] Sibata CH, Mota HC, Higgins PD (1990). Influence of hip prostheses on high energy photon dose distributions. Int J Radiat Oncol Biol Phys.

[B19] Tang X, Changran G (2013). Dosimetry effects of metal implants in patient body during radiation therapy. J Nanjing Univ Aeronautics Astronautics.

[B20] Thambi V, Murthy AK, Alder G, Kartha PK (1979). Dose perturbations resulting from gold fillings in patients with head and neck cancers. Int J Radiat Oncol Biol Phys.

[B21] Wieslander E, Knoos T (2003). Dose perturbation in the presence of metallic implants: treatment planning system versus Monte Carlo simulations. Phys Med Biol.

